# Age and sex differences in kidney microRNA expression during the life span of F344 rats

**DOI:** 10.1186/s13293-014-0019-1

**Published:** 2015-01-28

**Authors:** Joshua C Kwekel, Vikrant Vijay, Varsha G Desai, Carrie L Moland, James C Fuscoe

**Affiliations:** Division of Systems Biology, Personalized Medicine Branch, National Center for Toxicological Research, US Food and Drug Administration, 3900 NCTR Road, Jefferson, AR 72079 USA

**Keywords:** Kidney, miRNA expression, Sex, Age, Renal fibrosis, Renal inflammation, Biomarker

## Abstract

**Background:**

Growing evidence suggests that epigenetic mechanisms of gene regulation may play a role in susceptibilities to specific toxicities and adverse drug reactions. MiRNAs in particular have been shown to be important regulators in cancer and other diseases and show promise as predictive biomarkers for diagnosis and prognosis. In this study, we characterized the global kidney miRNA expression profile in untreated male and female F344 rats throughout the life span. These findings were correlated with sex-specific susceptibilities to adverse renal events, such as male-biased renal fibrosis and inflammation in old age.

**Methods:**

Kidney miRNA expression was examined in F344 rats at 2, 5, 6, 8, 15, 21, 78, and 104 weeks of age in both sexes using Agilent miRNA microarrays. Differential expression was determined using filtering criteria of ≥1.5 fold change and ANOVA or pairwise *t*-test (FDR <5%) to determine significant age and sex effects, respectively. Pathway analysis software was used to investigate the possible roles of these target genes in age- and sex-specific differences.

**Results:**

Three hundred eleven miRNAs were found to be expressed in at least one age and sex. Filtering criteria revealed 174 differentially expressed miRNAs in the kidney; 173 and 34 miRNAs exhibiting age and sex effects, respectively. Principal component analysis revealed age effects predominated over sex effects, with 2-week miRNA expression being much different from other ages. No significant sexually dimorphic miRNA expression was observed from 5 to 8 weeks, while the most differential expression (13 miRNAs) was observed at 21 weeks. Potential target genes of these differentially expressed miRNAs were identified.

**Conclusions:**

The expression of 56% of detected renal miRNAs was found to vary significantly with age and/or sex during the life span of F344 rats. Pathway analysis suggested that 2-week-expressed miRNAs may be related to organ and cellular development and proliferation pathways. Male-biased miRNA expression at older ages correlated with male-biased renal fibrosis and mononuclear cell infiltration. These miRNAs showed high representation in renal inflammation and nephritis pathways, and included miR-214, miR-130b, miR-150, miR-223, miR-142-5p, miR-185, and miR-296*. Analysis of kidney miRNA expression throughout the rat life span will improve the use of current and future renal biomarkers and inform our assessments of kidney injury and disease.

**Electronic supplementary material:**

The online version of this article (doi:10.1186/s13293-014-0019-1) contains supplementary material, which is available to authorized users.

## Background

Growing evidence suggests that epigenetic mechanisms of gene regulation may play a role in susceptibilities to specific renal diseases, toxicities, and adverse drug reactions. MicroRNAs (miRNAs) have been shown to be dysregulated in many renal pathologies [1,2]. For example, miR-193a has been identified as a potent regulator of focal segmental glomerulosclerosis (FSGS), a disease impacting the glomeruli [3]. In FSGS, podocyte function is severely disrupted by miR-193a inhibition of Wilms tumor 1/WT1, an important transcription factor in podocyte homeostasis. Furthermore, in diabetic conditions, expression levels of miR-195 and miR-29 have been shown to be elevated and reduced, respectively, in podocytes where they play a role in apoptosis and fibrosis [4]. The requirements of miRNAs in renal podocyte and glomerular development have also been demonstrated [5]. MiRNAs have been shown to have both positive and negative impact on kidney function. Complete oblation of miRNA function by deleting the miRNA processing gene Dicer in mouse podocytes reveals deficient podocyte homeostasis, reduced nephrogenesis, and other glomerular abnormalities that ultimately lead to lethality in young mice [6]. This suggests crucial roles of miRNA regulation in podocyte function that positively impact total kidney function. Similarly, miRNAs have been shown to function in response to drug-induced kidney injury. MiR-151 knockout mice have recently been used to demonstrate miR-151’s protection against cisplatin-induced kidney injury [7]. Further evidence pointed to miR-151’s protective effects being mediated via targeted suppression of c-Fos, a key regulator of apoptotic signaling [8]. These protective effects suggest miRNAs play a role in mediating key kidney functions; however, blocking miRNA function via Dicer knockout in proximal tubule cells showed protective effects against acute kidney injury [9]. Thus, mechanisms for both beneficial and deleterious roles of miRNA function in the kidney remain to be understood fully.

The rat model is often used in toxicology for assessment of drugs and other chemicals. However, little is known about the global expression patterns of miRNAs in various organs throughout the life span of the rat and the role they play in development and disease. MiRNAs show promise as predictive biomarkers for diagnosis and prognosis in a variety of tissues [10-14]. For example, nine miRNAs (miR-21, miR-20a, miR-146a, miR-199a-3p, miR-214, miR-192, miR-187, miR-805, and miR-194) have been identified in C57BL/6 mice as promising biomarkers of kidney injury after renal ischemia reperfusion injury (IRI) [15]. These miRNAs were shown to respond independent of lymphocyte infiltration, suggesting a kidney-specific response. Furthermore, three circulating plasma miRNAs (miR-16, miR-320, and miR-210) have been identified in acute kidney injury (AKI) patients as potential renal biomarkers [16]. MiR-210 in particular shows promise as a strong, independent prognostic biomarker of survival in AKI patients. Therapeutic targeting of miRNAs by antagomiRs to reduce adverse effects and disease states has also been successfully shown [17,18]. For example, anti-miR-192 has been used to decrease glomerular fibrosis in mouse models of diabetic nephropathy [19].

Sex differences in nondiabetic renal disease development and progression have been shown by several studies in humans [20-22] and in rodents [23]. Generally, males show greater susceptibility for various renal diseases (including adult FSGS, membranous nephropathy, and immunoglobulin A nephropathy) than females. Sex hormones appear to play a crucial role in imparting protective or predisposing effects [24]. The sex bias in susceptibility between males and females is less clear in diabetic renal disease [25,26]. Several reports suggest increased susceptibility of females to acute kidney injury and renal toxicity [27-29]. However, male susceptibilities to metal-induced nephrotoxicity and obesity-related adverse kidney effects have been demonstrated in *in vivo* models [30,31].

Advances have been made in characterizing cellular and molecular processes involved in various kidney functions; however, the molecular mechanisms responsible for age and sex differences and their potential impact on biomarker development are not well understood. Previous studies have demonstrated substantial sex differences in kidney messenger RNA (mRNA) expression in rodents [32,33], which may influence sexually dimorphic adverse events. Examination of miRNA regulation will further expand our understanding of the sex differences observed. While several miRNAs have previously been shown to exhibit sex specificity in expression and function in other tissues [34], there are no comprehensive studies that have examined miRNA sex bias in the kidney [35]. We investigated whether epigenetic mechanisms of gene regulation, such as changes in miRNA expression, are present in the kidney that may underlie age- and sex-specific susceptibilities to adverse renal events. Our findings show notable differences in miRNA expression that may inform our understanding of renal pathologies and the development of predictive biomarkers.

## Methods

### Animal study

Male and female Fischer 344 rats were obtained from the National Center for Toxicological Research rat breeding colony and maintained as previously described [33,36]. Rats used in the present study were sacrificed at 2, 5, 6, 8, 15, 21, 52, 78, and 104 weeks of age. Female animals were not synchronized for estrous cycle. Fischer 344 rats were used due to their frequent use for in-life bioassays in preclinical toxicology studies. The animal study was approved by the National Center for Toxicological Research (NCTR) Institutional Animal Care and Use Committee. Rats were housed two to a cage in polycarbonate cages with hardwood chip bedding; temperature was maintained at 23°C with a relative humidity of approximately 50%. Animals were euthanized by carbon dioxide asphyxiation and sacrificed at the same circadian time of day. Sections of the kidneys were collected from rats aged 52, 78, and 104 weeks for histological examination and placed in 10% neutral buffered formalin. Standard paraffin embedding, sectioning, and hematoxylin and eosin staining were performed before histopathological examination by a staff board-certified pathologist. Histological pathology scores range from 0 to 4, representing: none (0), minimal (1), mild (2), moderate (3), or marked (4) severity. Two endpoints were evaluated: renal fibrosis and renal mononuclear cell infiltration. All pathology findings are available in Additional file 1. A subset of the kidney pathology data was previously published [37]. Sex differences in histopathological severity scores were evaluated for statistical significance at 52, 78, and 104 weeks using a Mann–Whitney *U* test (also known as Wilcoxon Rank-Sum test). There were 8 to 16 animals per group and *p* values <0.05 were considered significant.

### RNA isolation

Whole kidneys from independent animals were ground to a powder in liquid nitrogen. Total RNA, including the small RNA fraction, was isolated from a 30-mg aliquot of ground kidney tissue powder and homogenized as previously described [33] using Qiagen miRNeasy Mini Kit (Qiagen Inc., Valencia, CA, USA) according to the manufacturer’s protocol. The yield of the extracted RNA was determined spectrophotometrically (Nanodrop-1000, Thermo Scientific, Wilmington, DE, USA) by measuring the optical density at 260 nm. RNA quality was evaluated using the RNA 6000 LabChip and Agilent 2100 Bioanalyzer (Agilent Technologies, Palo Alto, CA, USA). RNA samples with RNA integrity numbers (RINs) greater than 8.0 were used for microarray experiments with an average RIN of 8.5 for all samples.

### Microarray experiments

Genome-wide miRNA expression microarray experiments (*n* = 5 for 2-, 5-, 6-, 15-, 21-, and 78-week males and 6-, 15-, 78-, and 104-week females; *n* = 4 for 8- and 104-week males and 2-, 5-, 8-, and 21-week females) were completed for a total of 74 microarrays. Single color (Cy3) Agilent Rat 8 × 15 K miRNA microarrays and reagents were used according to the manufacturer’s protocols (Agilent Technologies, Santa Clara, CA, USA) using 100 ng of total RNA. An Agilent one-color spike-in kit was used as a positive control to measure both labeling and hybridization efficiency. Labeled probe was purified using BioRad Micro Bio-Spin 6 columns (BioRad Life Science Research, Hercules, CA, USA), according to the manufacturer’s protocol. Hybridized microarrays were scanned using the Agilent High Resolution C Scanner (Agilent Technologies, Santa Clara, CA, USA) according to the manufacturer’s protocol. The images were analyzed using Agilent’s Feature Extraction software. Expression data were deposited in Gene Expression Omnibus (Accession ID GSE64842).

### Microarray data analysis

Microarray TIFF images were analyzed using Agilent Feature Extraction (FE) software for preliminary quality control assessments which utilized labeling and hybridization spike-ins. The FE quality control evaluation metrics were within the ranges designated “good” or “excellent” for all microarrays. The raw (median) intensities were calculated in FE to generate a single value (total gene signal) for each miRNA. Individual array files (74 total) were uploaded in ArrayTrack [38,39], the Food and Drug Administration’s relational database for genomic data storage, processing, analysis, and visualization that was created at the NCTR. ArrayTrack is freely available at: http://www.fda.gov/ScienceResearch/BioinformaticsTools/Arraytrack/default.htm. A single file containing the raw intensities of miRNAs from all arrays was exported from ArrayTrack. An intensity value of 0.1 represented miRNAs that were not expressed. Intensity values of 0.1 were replaced by the minimum intensity value (1.39) across all the arrays. This file containing the raw intensities of all arrays was used as the input file for further data pre-processing in SAS 9.1.3 (SAS Institute Inc., Cary, NC, USA) to remove controls and target redundancy. Out of 15,744 array features, 2,204 features representing controls were removed, resulting in 13,540 features measuring sample miRNAs (677 unique miRNA × 20 replicates per miRNA). Redundant replicates (containing redundant values) were removed, resulting in a dataset containing 677 unique miRNAs. Next, those features which were not expressed (intensity = 1.39) in any of the arrays were discarded, leaving 311 unique features, which were expressed in at least one array. Raw intensity data for all arrays were transposed and transformed to log_2_ values, and normalization (75th percentile) was performed on the 311 miRNAs. Relative expression values (REVs) for each microarray feature/spot were calculated by taking individual animal normalized log2 intensity values divided by the averaged normalized intensities from all ages. Subsequently, a generalized linear model procedure (proc glm) in SAS was used to perform ANOVA to measure statistical significance of group differences using REVs as the dependent variable. Age effects were determined independently for males and females. Magnitude of group differences were calculated as the difference of log2 group means, and the anti-log of the resulting value equaled the fold change between groups. MiRNAs were considered differentially expressed by age when the ANOVA FDR was <5%, and age group differences were significant (*p* < 0.05) and showed ≥1.5 fold change difference between age groups. Sex effects were determined using pairwise *t*-test with FDR <5% and ≥1.5 fold change difference between sexes of the same age. There were 174 miRNAs that were differentially expressed by age or sex. Log2 REV values for all groups were subsequently exported as anti-log. Principal component analysis (PCA) and k-means clustering were performed in ArrayTrack on log2 normalized expression values. Group average REVs, principal component 1 (PC1) loading values, and k-means clusters for each differentially expressed miRNA (DEM) are shown in Additional file 2.

### miRNA functional analysis

Specific DEMs were analyzed using Ingenuity Pathway Analysis (IPA, http://www.ingenuity.com/) software. DEMs exhibiting an age effect were prioritized for pathway analysis using loading values from the PCA. Principal component 1 (PC1) represented primarily age-related differences in expression; therefore, the loading values for PC1 were rank ordered (Additional file 2). Those miRNAs having the most influence on PC1 variation ranked at the top and bottom of the distribution. Loading values with absolute value of ≥0.07 were included in gene lists for functional analysis. All DEM gene lists analyzed are shown in Additional file 3. Ingenuity pathways were considered significant if they exhibited a *p* value <1 × 10^−4^ and at least three molecules were represented. Top networks with scores >20 were also considered in functional analysis. The expression of three miRNAs associated with fibrosis (miR-142-5p, miR-150, miR-223) was correlated with histopathology fibrosis severity scores. Individual animal fibrosis severity scores of female and male animals (Additional file 1) from 78 and 104 weeks of age (*n* = 4 or 5 per group, total of 18 comparisons for each correlation) were matched with their respective miRNA expression data (normalized intensities), and Pearson correlation coefficients and respective *p* values were generated; *p* values <0.05 were considered significant. IPA infrequently associated a rat miRNA with a miRNA family gene name represented by a human miRNA of different name (e.g., rno-miR-363 is affiliated with miR-92a in gene list mapping); thus, miR-363 is associated with miR-92a in the Ingenuity knowledgebase for purposes of functional analysis.

### Taqman qPCR experiments

For each sample, 5 ng of total RNA was used in a 15 μl reverse transcription reaction using Taqman miRNA Reverse Transcription Kit (Life Technologies, Grand Island, NY, USA) and miRNA-specific primers according to the manufacturer’s instructions. The reverse transcription product (1.33 μl) was used in a 20 μl Taqman MicroRNA Taqman Assay reaction with predesigned miRNA-specific probes, according to the manufacturer’s instructions. Taqman PCR was conducted in MicroAmp Optical 384-well reaction plates (Applied Biosystems, Waltham, MA, USA) on an ABI 7900HT real-time PCR detection system. The expression level of each sample for each miRNA was standardized to U6 small nuclear RNA (snRNA), to control for differences in RNA loading, quality, and complementary DNA (cDNA) synthesis using the ΔΔCt method. For graphing purposes, the log2 relative expression levels are shown, such that the expression level of the mean expression for Taqman PCR (*n* = 5) and microarray data (*n* = 4 or 5) is equal to zero. A Pearson correlation coefficient (*R*) was calculated for each gene comparing Taqman and microarray relative expression with age.

## Results

### Sex- and age-associated renal histopathology

Histological examination of kidney tissues at 52, 78, and 104 weeks of age revealed moderate to marked mononuclear cell infiltration (average severity scores of 1, 1.9, and 2.5 at 52, 78, and 104 weeks, respectively) in males compared to only mild to moderate infiltration (average severity scores: 0.3, 1, and 1.6 at 52, 78, and 104 weeks, respectively) in females (Table 1 and Additional file 1). A similar trend was observed in the observations of renal fibrosis; males exhibited greater average severity than females at 52, 78, and 104 weeks (0.1, 0.5, and 1.5 in females and 0.7, 2.1, and 2.4 in males, respectively). Images illustrating these histopathological findings were previously published [33]. These results revealed sex-biased occurrences of renal pathologies associated with aging in male rats.

**Table 1:** **Histopathology average severity score**

**Age (weeks)**	**Fibrosis**			**Mononuclear cell infiltration**		
	**Female**	**Male**	***p*** **value**	**Female**	**Male**	***p*** **value**
*52*	0.1	0.7	0.025*	0.3	1	0.009*
*78*	0.5	2.11	0.001*	1	1.9	0.002*
*104*	1.5	2.43	0.014*	1.63	2.5	0.006*

*p < 0.05, Mann–Whitney *U* test comparing female and male groups at each age.

### Sex- and age-associated differential miRNA expression

**Figure 1:**
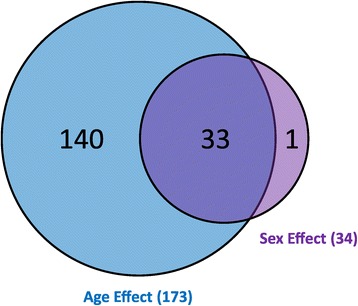
**Differential expression of miRNAs was calculated for age effects (ANOVA FDR <5%,** ***post hoc*** **test for multiple comparisons (** ***p*** **< 0.05), and fold change ≥1.5 between any two age groups; males and females analyzed independently) and sex effects (pairwise** ***t*** **-test, FDR <5% and ≥1.5 fold change between average female and male animals at each age).**

Of the 677 unique miRNAs present on the Agilent microarray, 311 were expressed at least once at any age in either sex. Those miRNAs whose expression changed with age were identified by ANOVA with FDR <5% with an additional requirement that the expression level difference between age groups be at least 1.5 fold. One hundred seventy-three miRNAs showed age-associated differential expression (Additional file 2); 131 miRNAs in females and 148 in males (approximately 75% overlap). Comparing male expression with female expression at each age using a *t*-test and FDR <5% showed sexual dimorphism in the expression of 34 miRNAs; 33 were differentially expressed by both age and sex. One DEM (miR-125a-5p) exhibited only a sex effect. Therefore, a total of 174 differentially expressed miRNAs (DEMs), by age or sex, were identified in the kidney (Figure 1).

**Figure 2:**
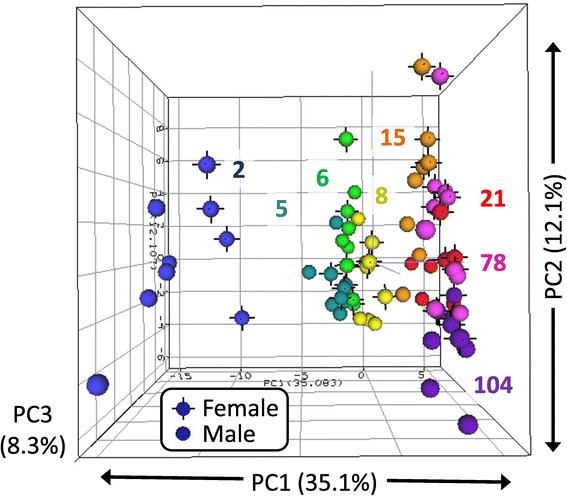
**One hundred seventy-four miRNAs meeting threshold criteria (ANOVA FDR <5%,** ***post hoc*** **test for multiple comparisons (** ***p*** **< 0.05), and fold change ≥1.5) for differential expression by age or sex were included in principal component analysis.** Each sphere represents the expression profile of one animal plotted in three-dimensional space according to the top three principal components. Spheres of the same color are animals of the same age group (weeks of age). Spheres with black vertices indicated females while those without represent males (*n* = 4 or 5).

These 174 DEMs were then used in PCA to identify relationships between the kidney miRNA profiles of animals within a group and between groups (Figure 2). PC1 appears to capture the variability in the expression data (35.1%) due to age, as age groups (distinguished by color) are distributed from young age to old age (left to right) along the PC1 axis. Variability in the expression data due to sex within age groups are not clearly observed except in 2-week animals, where females exhibit a slightly “older” profile than males. PC2 appears to represent interindividual variability of data from animals within the same age group. PC3 shows age differences that exist between the very young and very old compared to 5-, 6-, and 8-week-old animals. PC2 and PC3 accounted for 12.1% and 8.3% of the variability within the expression data, respectively. Within PC1, variability between animals of the same group appears to be less than variability between different age groups. That is, animals within age groups cluster more closely with each other than with other age groups. Among all groups, age showed a larger effect than sex, based upon larger variability represented by PC1, and no evident mapping of sex effect to the other principal components. Within PC1, the eight age groups clustered into three subgroups: 2-week animals, 5- to 8-week animals, and 15- to 104-week animals. However, animals from 15, 21, and 78 weeks of age did show slightly greater interindividual variability (primarily along the PC2 axis) than the other age groups while still maintaining general continuity between neighboring age groups along PC1. The 2-week-old animals showed the most separation from other age groups. However, all other age subgroups displayed a contiguous pattern, with neighboring age groups extending consecutively along the PC1 axis. Animals from the 5- to 8-week-old subgroup also showed separation from the 2-week and 15- to 104-week animals along the PC3 axis.

**Figure 3:**
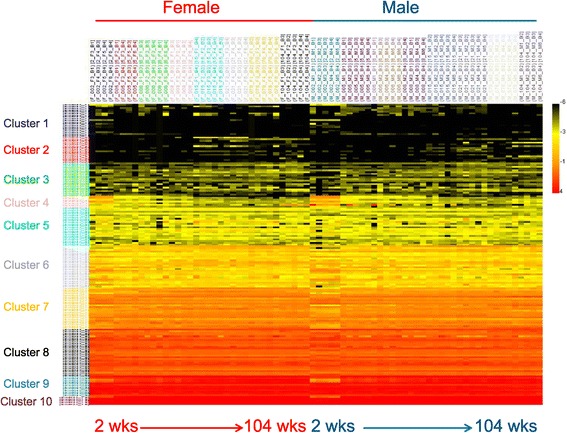
**Differentially expressed miRNAs (174 miRNAs) were clustered in 10 k-means clusters.** Ten clusters were chosen as an estimate of the number of large-scale expression patterns within the life span. As most miRNA expression changes are very subtle, miRNAs generally clustered according to normalized expression level.

To visualize the expression patterns of individual DEMs, k-means clustering was utilized to group miRNAs into common patterns (Figure 3). The number of k-means clusters was set at 10 as a modest estimation of the diversity present within the data. DEMs tended to cluster according to normalized intensity level; highly expressed miRNAs clustered with other highly expressed miRNAs and lowly expressed miRNAs with other low expressers. This clustering behavior likely occurred due to the relative stability of expression of all miRNAs throughout the life span. MiRNAs that are expressed at a certain level at an early age tend to maintain that expression level throughout the life span, in both sexes. Members of the let-7 family of miRNAs (7a, 7c, 7f, and 7b) exhibit the highest expression levels throughout the life span. Slight differences in expression are evident at 2 weeks for some miRNAs, especially those in clusters 4, 6, and 7.

**Figure 4:**
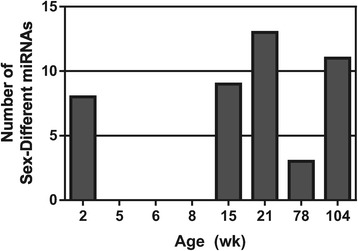
**The number of miRNAs exhibiting a sex difference in expression (pairwise** ***t*** **-test, FDR <5%, and fold change ≥1.5) was calculated for each age.** Groups of 5-, 6-, and 8-week-old rats exhibited no sex differences in miRNA expression, while 21-week-old rats showed the highest number.

The distribution of sex differences is not uniform across the life span (Figure 4). Eight miRNAs were expressed differently between males and females at two weeks of age, while none were found at 5, 6, and 8 weeks of age. Sex differences occurred from 15 to 104 weeks of age, with the most (13 miRNAs) observed at 21 weeks of age. Of the 34 miRNAs showing sex different expression, 17 exhibited female-biased expression (female expression > male expression, Table 2), while 17 exhibited male-biased expression (male expression > female expression, Table 3). The miRNAs showing the most consistent sex difference in expression (sex differences in at least three contiguous age groups) were miR-421* and miR-499. MiR-208* showed the highest fold change difference between sexes with female expression approximately 16 and 21-fold higher than male expression at 15 and 21 weeks of age, respectively. Other female-biased miRNAs showing large differences in expression included miR-139-3p (5-fold), miR-501 (6-fold), and miR-421* (2.6-fold). Several miRNAs also exhibited substantial male-biased expression, including miR-142-5p (7.8-fold), miR-130b (5.9-fold), and miR-206 (4.6-fold).

**Table 2:** **The 17 female-biased miRNAs**

**Name**	**Fold change (female/male)** ^**a**^								**Maximum sex difference (fold change)**	**Number of ages showing sex difference** ^**b**^	**Sex bias**
	**2 weeks**	**5 weeks**	**6 weeks**	**8 weeks**	**15 weeks**	**21 weeks**	**78 weeks**	**104 weeks**			
miR-421*	1.0	1.0	1.2	1.4	*2.1*	*1.6*	*2.6*	*2.0*	2.6	4	F > M
miR-499	1.6	1.0	1.2	1.4	*2.2*	*2.4*	*1.9*	1.5	2.4	3	F > M
miR-208*	1.1	1.3	1.0	2.0	*16.3*	*21.8*	2.0	1.2	21.8	2	F > M
miR-139-3p	0.7	1.9	0.8	3.5	1.0	*5.0*	1.6	*4.2*	5.0	2	F > M
miR-501	6.0	0.6	1.5	0.6	4.4	0.4	*7.3*	0.8	7.3	1	F > M
miR-29c	*2.0*	1.0	0.9	0.9	1.0	0.9	1.1	1.2	2.0	1	F > M
miR-33	*2.0*	1.0	1.0	0.7	0.7	0.7	1.1	1.1	2.0	1	F > M
miR-29b	*2.0*	1.1	1.0	0.9	1.0	0.9	1.1	1.3	2.0	1	F > M
miR-183	0.9	0.9	1.6	1.7	*1.9*	1.4	1.1	0.8	1.9	1	F > M
miR-375	1.6	1.0	1.0	1.4	*1.8*	1.5	1.4	1.1	1.8	1	F > M
miR-29a	*1.7*	1.1	0.9	1.0	1.1	0.9	1.2	1.1	1.7	1	F > M
miR-3545-3p	*1.6*	1.1	0.9	0.8	0.9	1.0	1.2	1.6	1.6	1	F > M
miR-34c	1.2	0.9	1.5	1.7	*1.6*	1.5	0.5	0.6	1.6	1	F > M
miR-129-2*	1.4	1.1	0.8	1.8	1.6	1.2	1.7	*1.6*	1.6	1	F > M
miR-192	1.4	0.9	0.9	0.9	1.0	0.9	1.4	*1.6*	1.6	1	F > M
miR-29c*	*1.6*	1.0	0.9	1.0	1.1	0.9	1.2	1.3	1.6	1	F > M
miR-210	1.1	1.1	1.0	1.1	*1.5*	1.4	1.5	1.5	1.5	1	F > M

^a^Italicized values indicate differential expression by sex.

^b^Sex difference according to pairwise *t*-test, FDR 5%, and fold change ≥1.5.

**Table 3:** **The 17 male-biased miRNAs**

**Name**	**Fold change (male/female)** ^**a**^								**Maximum sex difference (fold change)**	**Number of ages showing sex difference** ^**b**^	**Sex bias**
	**2 weeks**	**5 weeks**	**6 weeks**	**8 weeks**	**15 weeks**	**21 weeks**	**78 weeks**	**104 weeks**			
miR-130b	*1.5*	1.0	1.2	1.6	3.1	2.0	2.3	*5.9*	5.9	2	M > F
miR-204	1.1	1.0	1.3	1.3	*1.6*	*2.0*	1.1	1.2	2.0	2	M > F
miR-142-5p	0.9	0.7	1.0	0.9	1.2	4.0	3.3	*7.8*	7.8	1	M > F
miR-206	0.9	0.7	0.9	1.3	2.1	*4.6*	1.1	0.7	4.6	1	M > F
miR-142-3p	0.6	1.0	1.0	1.4	*1.5*	1.4	2.4	*2.1*	2.1	1	M > F
miR-135a	0.7	0.8	1.0	0.9	1.6	*2.1*	0.7	0.8	2.1	1	M > F
miR-223	0.9	1.1	1.0	1.0	1.1	1.3	1.6	*1.9*	1.9	1	M > F
miR-296*	*1.7*	0.7	1.0	0.9	0.9	0.5	0.7	0.7	1.7	1	M > F
miR-505	1.2	1.1	1.3	1.0	1.2	*1.6*	1.1	1.1	1.6	1	M > F
miR-150	0.9	0.9	1.0	0.9	1.0	1.0	1.2	*1.6*	1.6	1	M > F
miR-199a-5p	1.1	1.0	1.0	1.0	0.9	1.0	1.4	*1.6*	1.6	1	M > F
miR-22*	1.0	1.1	1.1	1.3	1.3	*1.6*	1.0	1.1	1.6	1	M > F
miR-455*	1.1	1.1	1.1	1.2	1.6	*1.5*	1.0	1.0	1.5	1	M > F
miR-125a-5p	1.2	1.0	1.0	1.1	1.2	*1.5*	0.9	1.0	1.5	1	M > F
miR-214	1.2	1.0	1.0	0.9	1.0	1.1	1.2	*1.5*	1.5	1	M > F
miR-185	0.9	1.0	1.1	1.2	1.2	*1.5*	0.9	0.9	1.5	1	M > F
miR-181b	1.2	0.9	1.2	1.1	1.2	*1.5*	1.1	1.0	1.5	1	M > F

^a^Italicized values indicate differential expression by sex.

^b^Sex difference according to pairwise *t*-test, FDR 5%, and fold change ≥1.5.

**Figure 5:**
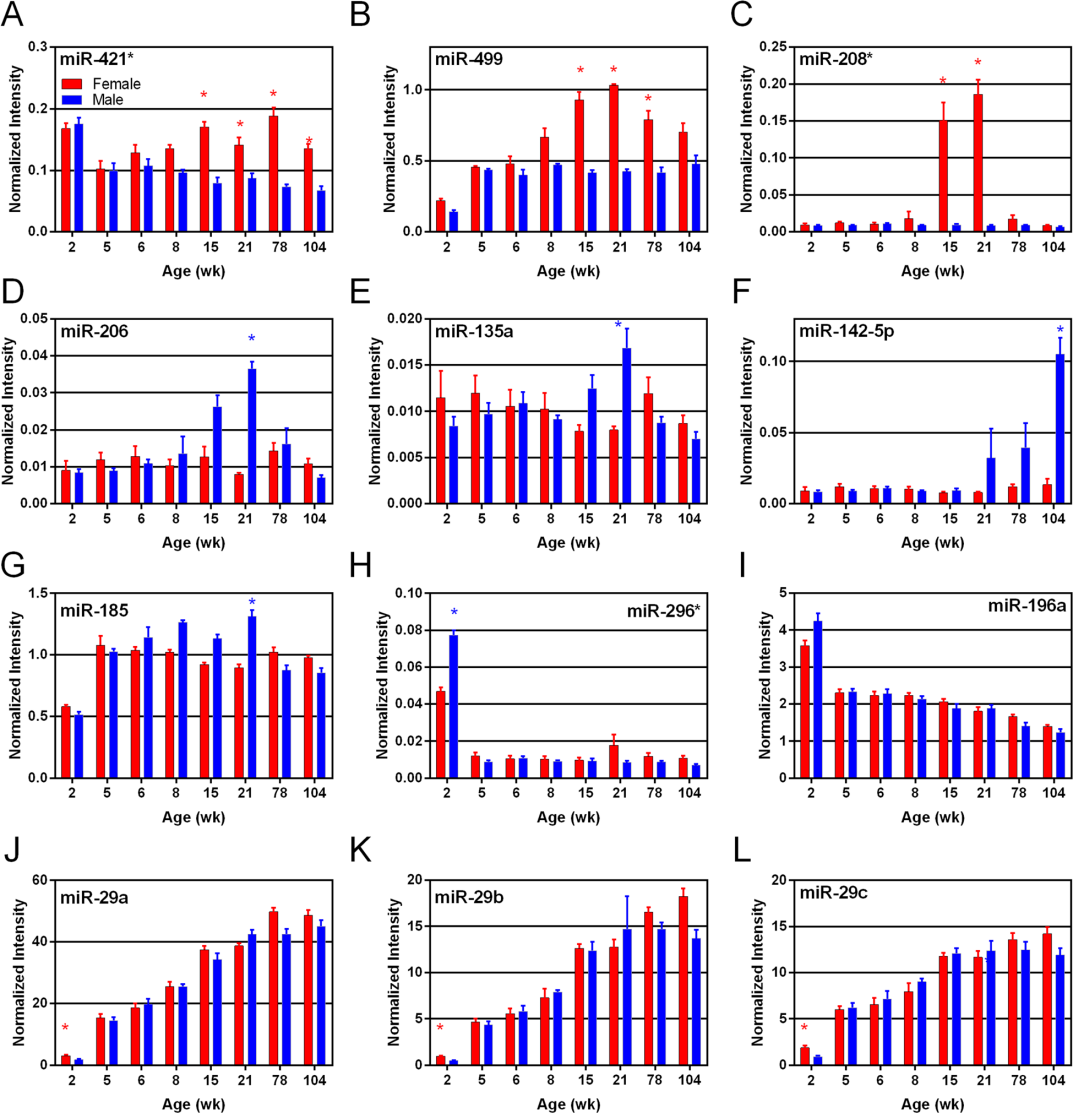
**Plots of individual miRNAs (microarray data) exhibiting sex and age differences in expression are shown (** ***n*** **= 4 or 5). A**, **B**, and **C** show miRNAs exhibiting female-biased miRNA expression; **D**, **E**, **F**, and **G** show male-biased miRNAs; **H** and **I** show young age expressed miRNAs; and **J**, **K**, and **L** show old age expressed miRNAs. Bars represent normalized intensity values per age group; blue and red colors represent male and female data, respectively. Asterisks indicate ages where sex difference in expression is statistically significant (*t*-test, FDR <5%).

**Figure 6:**
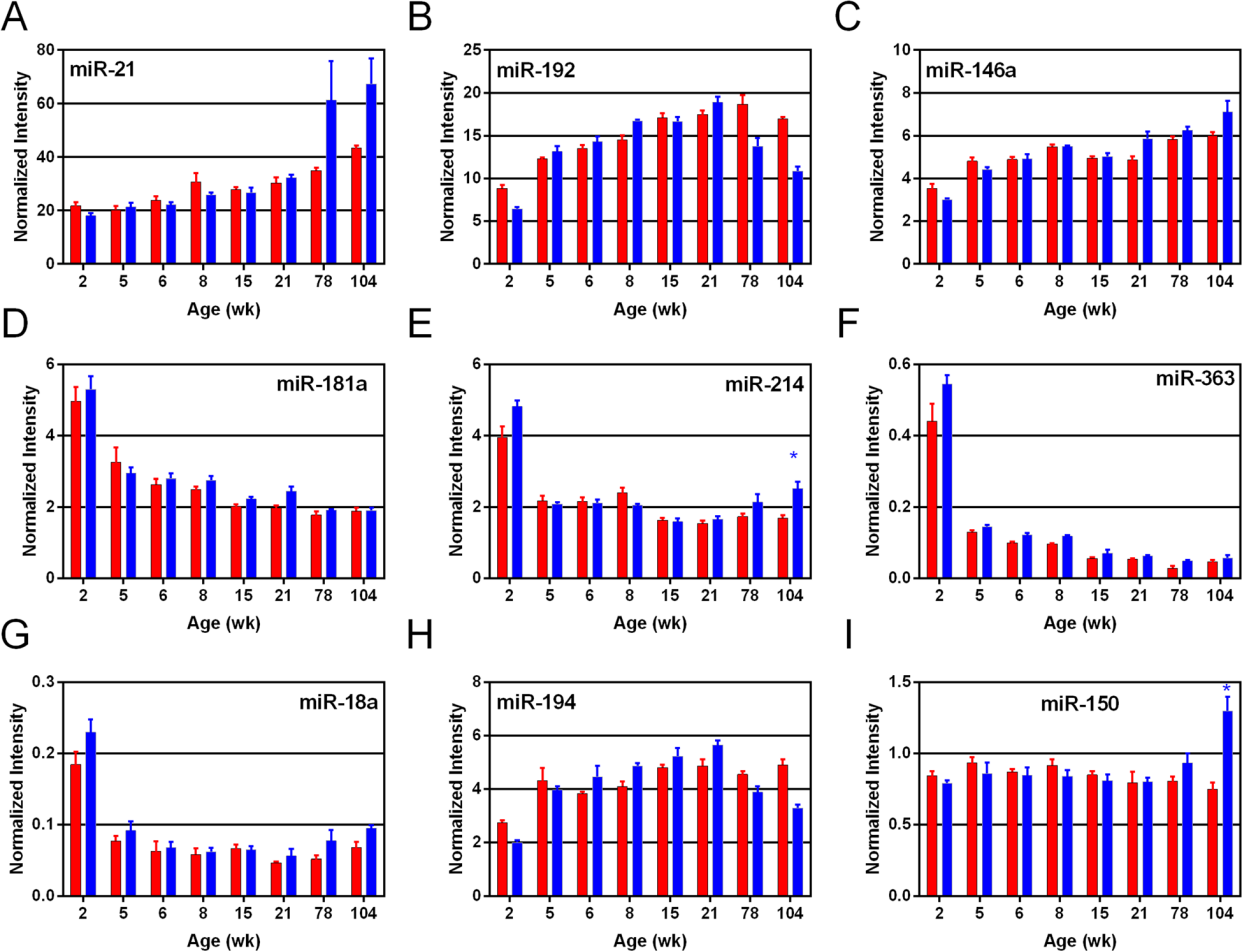
**Plots of individual miRNAs (microarray data) exhibiting sex and age differences in expression are shown (** ***n*** **= 4 or 5). A**, **B**, **C**, **H**, and **I** show middle to late age expressed miRNAs; **D**, **E**, **F**, and **G** show young age expressed miRNAs. **E** and **I** also show miRNAs exhibiting significant sex differences. Bars represent normalized intensity values per age group; blue and red colors represent male and female data, respectively. Asterisks indicate ages where sex difference in expression is statistically significant (*t*-test, FDR <5%).

Examples of the expression profiles of miRNAs that change with age and/or sex are shown in Figures 5 and 6. Female-biased miRNA expression is illustrated by miR-421*, miR-499, and miR-208* (Figure 5A-C), all of which showed female-biased expression at 15 and 21 weeks. MiR-499 also exhibited female bias at 78 weeks of age and miR-421* at 78 and 104 weeks of age. Male-biased miRNA expression was observed for miR-206 and miR-135a (Figure 5D-E), both of which showed higher expression in males at 21 weeks of age. Male-biased expression at older ages was observed for miR-142-5p (Figure 5F) with significant difference at 104 weeks of age. Age-specific changes in miRNA expression were found for miR-296*, miR-196a (Figure 5H-I), miR-181a, miR-214, miR-363, and miR-18a (Figure 6D-G) which showed high expression at 2 weeks of age in both sexes, followed by low and decreasing expression at all subsequent ages. The miRNAs that showed highest expression during middle or young adult ages (15 and 21 weeks) were miR-192 and miR-194 (Figure 6B,H), both of which show steady increase in expression at early ages, that peak at 15 and 21 weeks of age, before decreasing at older ages. MiRNAs exhibiting old-age expression included miR-21, miR-146a (Figure 6A,C), and members of the miR-29 family (miRa/b/c), which exhibit low expression at young age with increasing expression at older ages (78 and 104 weeks) (Figure 5J-L).

**Figure 7:**
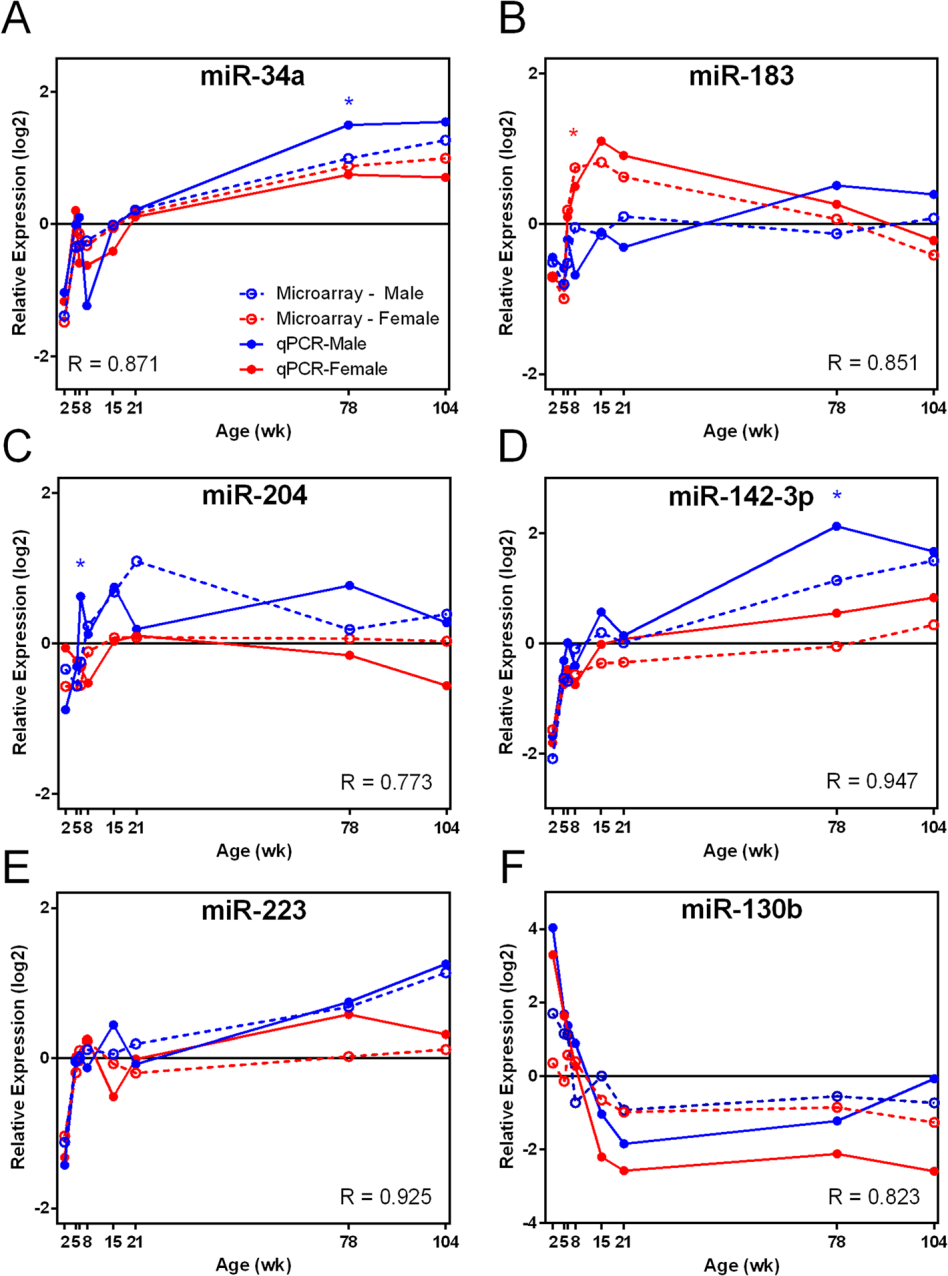
**Select miRNAs exhibiting sex and age differences were verified by Taqman qPCR. A**, **E**, and **F** show age related changes in miRNA expression; **B**, **C**, and **D** show sex related changes in expression. MiRNA expression relative to the mean expression level (relative expression log2) is shown on Y axis. Microarray and qPCR data are represented by dotted and solid lines, respectively. Male and female are indicated by blue and red colors, respectively. The Pearson correlation coefficient (*R*) was calculated between microarray and qPCR data for each miRNA. Asterisks indicate ages where statistical difference in expression between sexes exists according to pairwise *t*-test (*p* < 0.05) for each age in qPCR data.

Six miRNAs were selected for verification by Taqman quantitative PCR, and the expression profiles are shown in Figure 7 along with the profiles from microarray analysis. Statistically significant sex differences were confirmed for miR-183, miR-204, and miR-142-3p (Figure 7B-D). Significant age differences in the expression of miR-34a, miR-223, and miR-130b (Figure 7A,E,F) were confirmed by qPCR. Pearson correlation coefficients were calculated for each miRNA, comparing qPCR and microarray data. Correlations ranged from *r* = 0.77 to *r* = 0.95 with an average correlation of 0.87, suggesting good agreement between the two methods in measuring miRNA expression.

### Functional analysis of differentially expressed miRNAs

MiRNAs displaying the most prominent age and sex effects were further examined for functional analysis to determine potential biological roles these miRNAs might be playing in the kidney. Prominent age-affected miRNAs were selected using PCA loading values. Examination of Figure 2 suggests that PC1 represents age-associated differences in miRNA expression among the sample groups. Those miRNAs with the highest PC1 loading values (absolute values ≥0.07) have the largest influence on the age-related profile and were thus selected for functional analysis. The PC1 loading values for all 174 differentially expressed miRNAs are shown in Additional file 2. Thirty-nine miRNAs had loading values > |0.07|. The miRNAs with the most negative loading values in PC1 were those that were generally most highly expressed in 2-week-old rats with decreasing or no expression at subsequent ages. Seventeen DEMs exhibited this pattern and thus were analyzed as young age-expressed miRNAs. Conversely, positive loading values in PC1 generally represented miRNAs showing increasing expression with age, with highest expression at 78 and 104 weeks of age. Twenty-two DEMs represented old age-expressed miRNAs. Thus, the young age effect (2-week-old expression, 17 miRNAs) and old age effect (78- and 104-week-old expression, 22 miRNAs) miRNAs were analyzed using Ingenuity Core Analysis (Additional file 3, Table 4). MiRNAs associated with young age expression showed the highest enrichment for pathways involved in renal inflammation/nephritis (miR-130b, miR-363, miR-296*) and cancer (miR-214, miR-130b, miR-18a, miR-181a, miR-363, miR-196a). In total, 15 IPA subcategories including the term “cancer” showed significant enrichment (at least three molecules with *p* value <1 × 10^−4^) among young age-expressed miRNAs. Old age-associated miRNAs showed enrichment in pathways related to endocrine system disorders (miR-129-1, miR-375, miR-223, miR-664, miR-29b, miR-34a), cancer (miR-223, miR-29b, miR-375, miR-96), and cellular movement/invasion of cells (miR-29b, miR-29c, miR-7a, miR-96, miR-34a, miR-375).

**Table 4:** **Pathway analysis results**

**Analysis**	**Gene list** ^**a**^	**Pathway analysis results** ^**b**^	**Number of miRNAs**	***p*** **value/score**
Age effects	Young age expression (17 miRNAs)	*Top tox func*: renal inflammation/renal nephritis	3	3.68E-05
		*Disease and bio func*: organismal inj./abnorm. (nonobstruct azoospermia)	5	2.55E-08
		cancer (cervical squamous cell carcinoma)	6	1.14E-07
		*Top network*: organismal inj./abnorm., repro. syst. dis., cancer	10	27
	Old age expression (22 miRNAs)	*Top tox func*: N/A	-	-
		*Disease and bio func*: endocrine syst. dis. (non-insul-dep. diab. mellitus)	6	1.13E-07
		cancer (pancreatic ductal adenocarcinoma)	4	1.16E-07
		cellular movement (invasion of cells)	6	6.20E-05
		*Top network*: cancer, endocrine syst. dis, gastrointestinal dis.	9	22
Sex effects	Female > male (17 miRNAs)	*Top tox func*: N/A	-	-
		*Disease and bio func*: cancer (uterine endometrioid carcinoma)	4	1.19E-09
		organismal inj. and abnorm (nonobstruct azoospermia)	5	9.28E-09
		endocrine syst disorder (non-insulin-dep diabetes mellitus)	6	1.11E-08
		*Top network*: organismal injury, repro syst dis, cell death and survival	10	27
	Male > female (17 miRNAs)	*Top tox func*: renal inflammation/renal nephritis (lupus nephritis)	6	1.93E-10
		*Disease and bio func*: cancer (cervical squamous cell carcinoma)	9	1.61E-18
		hematological dis (nasal natural killer cell lymph)	5	1.87E-10
		*Top network*: cancer, organismal inj & abnorm, repro system disorder	11	28

^a^Gene lists are available in Additional file 3.

^b^IPA was used to identify significantly enriched pathways.

In a similar manner, the predominant sex-affected DEMs were selected based upon differential expression between sexes at any age (FC > 1.5, *t*-test and FDR <5%). Sex-biased miRNAs were represented by 17 female-biased and 17 male-biased miRNAs from Tables 2 and 3. Female-biased miRNAs were characterized by enrichment for pathways associated with organismal injury and abnormalities/nonobstructive azoospermia (miR-29b, miR-29c, miR-33, miR-499), endocrine system disorders (miR-129, miR-183-3p, miR-29b, miR-375, miR-33), and cancer (miR-183-5p, miR-192, miR-210). Male-biased miRNA expression was associated with pathways related to cancer (miR-130b, miR-214, miR-181b, miR-199a, miR-150, miR-135a, miR-142-3p, miR-142-5p, miR-185), hematological disease (miR-22*, miR-142-3p, miR-142-5p, miR-150, miR-181b), and renal inflammation/nephritis (miR-130b, miR-223, miR-150, miR-142-5p, miR-296*, miR-185-3p) (Additional file 2).

## Discussion

### Age effects more prevalent than sex effects during rat life span

The global evaluation of miRNAs in the rat kidney revealed 311 miRNAs expressed at one or more ages in either sex during the rat life span. This is roughly half of the total 677 miRNAs present on the Agilent rat microarray, suggesting a substantial role for miRNA regulation of gene expression within the kidney. Because the samples were derived from whole, ground kidneys, it is not possible to assign expression of these miRNAs to specific substructures of the kidney, such as the medulla or cortex. Follow-up studies will be necessary to identify the different kidney cell types making contributions to the expression profiles. Of the 311 expressed miRNAs, 174 of them (56%) showed significant change in expression between ages or sexes (at least a 1.5 fold difference in expression and *p* value <0.05). These changes in miRNA expression manifested themselves generally in three age subgroups according to PCA, consisting of young, middle, and old age groups. The young subgroup (2 weeks old) showed the largest difference from all other subgroups and also showed slight difference between males and females, whereas all other subgroups showed little to no sex difference in miRNA expression. These findings suggest that in our study, age influences basal miRNA expression more than sex for most miRNAs expressed in the kidney. This is also supported by the fact that the number of DEMs exhibiting sex differences (34 DEMs) was lower than those showing age differences (173 DEMs). Despite the number of miRNAs showing differential expression, k-means cluster analysis revealed that the expression profiles generally grouped according to expression level (Figure 3). MiRNAs of a certain expression level typically maintained that level for the majority of the life span in both sexes, showing a relatively constant expression pattern compared to differentially expressed genes (mRNA) [33]. Evaluation of how sex-biased miRNAs are distributed across the age groups (Figure 4) showed a noticeable gap in sex-biased expression between 2 and 15 weeks of age; ages at which large-scale pubertal developmental changes are taking place in the rat. This suggests that the more subtle or fine-tuned regulation by miRNAs of gene expression may not be involved in sexually dimorphic expression changes during this time.

### Functional analysis reveals overlapping and redundant pathways

Identifying the miRNAs that display age and sex differences was a crucial first step in elucidating the potential roles that miRNAs may play in sexually dimorphic and age-related susceptibilities. However, the specific metabolic pathways controlled by these DEMs in the kidney are not well understood [40,41]. In general, functional analysis (Ingenuity) of the most predominant miRNAs showing age and sex-specific effects resulted in pathways and functional categories with notable overlap between the various age and sex analysis groups (Table 4). For example, cervical carcinoma or nonobstructive azoospermia pathways occurred among the top findings in three of the four analysis groups. The same miRNAs were frequently found to populate several pathways with few pathways exhibiting distinct, non-overlapping miRNA lists. For example, miR-130b was found in pathways related to cancer, renal inflammation, and organismal injury and abnormalities, while miR-150 was found in both cancer and renal inflammation pathways. This may be explained by the current, relatively immature status of miRNA functional analysis databases and tools compared to those available for gene expression (mRNA). Despite the presence of highly ranked functional categories (according to Ingenuity *p* value and number of member miRNAs) that are not readily interpretable in the context of the kidney (e.g. cervical carcinoma, nonobstructive azoospermia), several trends that relate to kidney physiology were evident in the results and are discussed below.

### Young rats express proliferation-related miRNAs

One trend related to age effects was observed in the young age expression group (17 miRNAs expressed predominantly at 2 weeks of age, Additional file 3). Functional analysis for the young age miRNAs revealed several pathways related to hyperplasia, proliferation and cancer being over represented. More than 15 cancer-related pathways ranked among the top findings for the young age group and included common miRNAs: miR-363, miR-181a, miR-130b, and miR-18a (Figures 5 and 6). It is likely that the “cancer”-related categories from the IPA analysis are representative of cell processes related to hypertrophy, proliferation, or differentiation as opposed to a disease state; especially in such young animals (2 weeks old). MiR-363 is a member of the miR-92a family and its 2-week expression is consistent with its role in the formation of vascular endothelial cells during mammalian organ development [42]. MiR-181a and miR-130b have also been shown to be involved in endothelial cell proliferation [43]. MiR-18a and miR-92a are members of the miR-17/92 cluster, the first characterized oncogenic miRNA cluster, called *oncomir-1* [44]. The cluster’s activities in cell proliferation and tumorigenesis are well characterized. MiR-18a targets Smad2 and Smad4, two members of the TGF-*β* signaling pathway. The expression of these miRNAs early in the rat life span (2 weeks) suggests overlapping roles in regulating cellular proliferation and differentiation during early kidney development at 2 weeks of age that were not needed at subsequent ages, as their expression decreases substantially.

### Old age-related miRNA expression

A second age-related effect involved the old age expression group (22 miRNAs with increasing expression levels at later ages, Additional file 3). DEMs within this group had the most influence in old age expression, based upon highly ranked PC1 loading values. The miRNAs showing the highest expression within this group were three members of the miR-29 family (miR-29b, miR-29a, and miR-29c). Each of these three showed increasing expression with age in both sexes. MiR-29 has previously been shown to play a protective role in attenuating CCl4-induced liver fibrosis [45]. However, renal fibrosis observed in histopathology sections in the current study (Table 1) was more apparent in male rats compared to females, suggesting some other, possibly tissue-specific, mechanisms. MiR-29b has also been shown to negatively regulate DNA methylation via its direct targeting of DNMT3A and DNMT3B, which are involved in *de novo* DNA methylation [46]. MiR-29 family repression of DNA methylation activities in older animals would suggest multiple levels of epigenetic regulation. A second age-affected DEM among the highest PC1 loading values is miR-34a, which shows increasing expression with age in both sexes (Figure 7). MiR-34a is known to target p53-related apoptosis signaling and has been previously shown to be upregulated in the kidney in conjunction with cisplatin-induced renal toxicity [47]. It is not clear how the age-related increase in miR-34a may influence susceptibility, but its role in cell death may be a key. Functional analysis of old age-expressed miRNAs found pathways related to cellular movement and invasion of cells (Table 4), which also relates to the histopathology findings of renal mononuclear cell infiltration (Table 1), likely in conjunction with immune response to increasing fibrosis with old age. In general, the age-specific expression of miRNAs in the rat kidney appears to represent pathways reflecting the developmental and aging activities present at young and old age, respectively.

### Female-biased miRNA expression predominant in young adult animals

MiRNAs that were differentially expressed between the sexes were grouped according to either female-biased or male-biased miRNAs. Those DEMs exhibiting female bias (17 miRNAs, Additional file 3) included miRNAs showing the highest fold-change differences between sexes (miR-208*, with 21-fold change F > M) and most prolonged, sex-biased expression (miR-421*, at 15, 21, 78, and 104 weeks). Several female-biased miRNAs exhibited both age and sex effects and have been associated with proliferation, cell death, and immune function in various cancers. For example, both miR-499 and miR-421*, which showed the most female-biased expression at 15, 21, and 78 weeks of age (Figure 5), have been shown to regulate proliferation and apoptosis via different cellular pathways [48,49]. MiR-421* showed significant female-biased expression over the longest timespan in the kidney (15–104 weeks of age) (Figure 5). Interestingly, this miRNA maps to the X chromosome and has been associated with X-chromosome activation and imprinting [50]. MiR-183 also showed female-biased expression during middle age (Figure 7) and has been proposed as a promising biomarker for early detection and prognosis of certain cancers including colorectal and prostate [51]. Both miR-375 and miR-223 play roles in regulating inflammation [52] but exhibited very different, sex-biased expression in the kidney, with miR-375 showing female-biased expression at 15 weeks of age (Table 2) and miR-223 showing male-biased expression at old age (Table 3 and Figure 7). Female-biased miRNAs feature some of the most promising candidates (miR-421*, miR-499, miR-208*) to further investigate the potential impact of sex-biased expression of miRNAs in the kidney.

### Male-biased expression of inflammation/fibrosis-related miRNAs in old age

Sex effects in kidney miRNA expression were also found to exhibit an equivalent number of male-biased miRNAs (17 miRNAs, Additional file 3). Functional analysis of male-biased DEMs showed conspicuous representation of pathways related to renal inflammation and renal nephritis (six miRNAs, *p* value 1.93 × 10^−10^). These six miRNAs are miR-130b, miR-296*, miR-223, miR-142-5p, miR-185, and miR-150. Three of these six molecules exhibited a pattern of increasing expression with age (miR-223, miR-150, miR-142-5p). This suggests that renal inflammation/nephritis pathways exhibit male-biased patterns that increase with age. This finding agrees with the male-biased fibrosis and mononuclear cell infiltration pattern found in male histology sections (Table 1). Individual animal kidney fibrosis severity scores correlated positively and significantly (*p* < 0.05) with individual miR-142-5p (*R* = 0.526), miR-150 (*R* = 0.567), and miR-223 (*R* = 0.724) expression from those animals at 78 and 104 weeks of age. Furthermore, miR-142-5p and miR-223, both male-biased DEMs (Figures 5 and 7), have been previously implicated in fibrosis pathways [53], highlighting agreement regarding the functional roles of these miRNAs. However, the finding that the renal inflammation/nephritis pathway was also represented in the young age gene list (17 miRNAs, high expression at 2 weeks with decreasing expression at subsequent ages) seems to be confounding with the hypothesis of old age activity in this pathway. Nevertheless, the possibility of both positive and negative activities among these pathway members could account for the different expression patterns. One of the most notable male-biased miRNAs, miR-206 (Figure 5), has been shown to directly target cyclin D1, a key regulator of cell cycle control [54]. Another male-biased miRNA, miR-204 (Figure 7), has been shown to directly target Sirt1, a key regulator of various metabolic processes, including resistance to oxidative stress and fibrosis [55]. Male-biased, age-associated increases in kidney fibrosis and mononuclear cell infiltration were observed by analysis of tissue sections (Table 1; Additional file 1). Male animals had fibrosis and mononuclear cell infiltration severity scores greater than 1.5 times those of females at 78 and 104 weeks of age; thus, the male-biased increase in expression of miR-204 at prior ages (15 and 21 weeks) may be linked, via downregulation of Sirt1, to the age-associated kidney fibrosis seen in our study. MiR-204-induced reduction of Sirt1 protein may be expected to make the kidney more susceptible to oxidative injury. In fact, Sirt1 expression appears to protect against ischemia/reperfusion-induced acute kidney injury [56] and unilateral ureteral obstruction induced renal injury [57] in mouse models. So, while male-biased miRNAs did not exhibit the highest or most prolonged sex differences in expression (as in the female-biased miRNAs), their sex-biased expression could be connected to physiologically relevant endpoints (fibrosis, inflammation).

### Potential roles of kidney miRNA as biomarkers of disease and toxicity

MiRNAs are being examined in various pathologies as biomarkers, as well as for disease therapy [58-62], although limited information is available at this time for the kidney [63]. The miRNA showing the greatest fold-change difference between the sexes was miR-208* which showed approximately 21-fold higher expression in females compared to males at 21 weeks of age (Table 2 and Figure 5). MiR-208*, as well as miR-499 (Figure 5), which showed a 2.5 fold higher level in females than males at 21 weeks of age, have been predominantly characterized in cardiac tissue, as promising biomarkers of cardiotoxicity [64]. Their role in the kidney, and particularly their notable sex-difference in expression, requires further investigation. MiR-210, which exhibits female-biased expression (1.5 fold at 15 weeks), has been identified as a potential biomarker in plasma samples for predicting survivability in AKI patients [16]. Furthermore, elevated miR-210 expression in renal carcinoma tissue is predictive of better clinical outcomes for clear cell renal cell cancer patients [65]. In our study, miR-210 showed slight but statistically significant female bias in expression, with females showing 1.5 fold higher expression than males at 15 weeks of age (Table 2). This sex difference may contribute a slight protective effect for females against AKI or certain renal cancers, but further study is necessary.

A previous report characterized an miRNA expression signature, consisting of nine miRNAs, that was specific to ischemic reperfusion injury [15]. Of the nine miRNAs in this IRI-specific expression signature, six showed differential expression in our study (four of which exhibited increasing expression with age, including miR-21, miR-146a, miR-192, and miR-194) (Figure 6). Although miR-21 exhibited a conspicuous pattern of higher expression in males compared to females at 78 and 104 weeks (both ages exhibiting >1.5 fold difference), the sex difference was not statistically significant. However, miR-214 exhibited sex-biased increase in expression at 104 weeks, showing 1.5 fold higher expression in males than in females. These findings suggest that age and sex differences in expression may need to be considered when utilizing such classifiers.

## Conclusions

The specific sex- and age-associated miRNAs identified in the kidney of a rat model is an important first step in understanding the role that epigenetic mechanisms of gene regulation might play in sex- and age-associated susceptibilities. To our knowledge, this is the first presentation of global profiles of kidney miRNA expression throughout the entire rat life span in both sexes. Several lines of evidence, most notably the male-biased expression of miRNAs and their linkage to inflammation and fibrosis pathways, point to substantial involvement of age- and sex-related miRNAs in the male-biased renal fibrosis and mononuclear cell infiltration observed in our study. Additionally, developmental roles for miRNAs expressed predominantly in very young (2 week) animals are suggested by pathways related to cancer and proliferation. Further work will be required to elucidate the biological impact of the sex differences observed. However, the presence of clear age and sex differences among many kidney miRNAs promises deeper understanding of kidney biology and potential renal biomarkers.

## Additional files

Additional file 1:
**Histopathology report.** Histopathological findings regarding kidney fibrosis and mononuclear cell infiltration for individual animals at 52, 78, and 104 weeks of age. Additional file 2:
**One hundred seventy-four differentially expressed miRNAs.** Relative expression values for all differentially expressed miRNAs by age and/or sex; including maximum age group difference, number of significant age differences, PC1 loading values, and k-means cluster assignments. Table is sorted by absolute value of PC1 loading values. Additional file 3:
**Pathway analysis miRNA lists.** Four lists of miRNAs used for pathway analysis in IPA for evaluating age effects (young age-expressed and old age-expressed miRNAs) and sex effects (female-biased and male-biased miRNAs).

## Abbreviations

AKI: acute kidney injury
DEMs: differentially expressed microRNAs
F344: Fischer 344
FSGS: focal segmental glomerulosclerosis
FDR: false discovery rate
FE: feature extraction
IPA: Ingenuity Pathway Analysis
IRI: ischemic reperfusion injury
miRNA: microRNA
NCBI: National Center for Biotechnology Information
PCA: principal component analysis
PC: principal component
REV: relative expression value
RIN: RNA integrity number
